# Antioxidant Peptides from *Hizikia fusiformis*: A Study of the Preparation, Identification, Molecular Docking, and Cytoprotective Function of H_2_O_2_-Damaged A549 Cells by Regulating the Keap1/Nrf2 Pathway

**DOI:** 10.3390/foods14030400

**Published:** 2025-01-26

**Authors:** Shang Lv, Bin Hu, Su-Zhen Ran, Min Zhang, Chang-Feng Chi, Bin Wang

**Affiliations:** 1 National and Provincial Joint Laboratory of Exploration and Utilization of Marine Aquatic Genetic Resources, National Engineering Research Center of Marine Facilities Aquaculture, School of Marine Science and Technology, Zhejiang Ocean University, Zhoushan 316022, China; 2 Zhejiang Provincial Engineering Technology Research Center of Marine Biomedical Products, School of Food and Pharmacy, Zhejiang Ocean University, Zhoushan 316022, China; 3 School of Foundation Studies, Zhejiang Pharmaceutical University, Ningbo 316022, China

**Keywords:** hijiki (*Hizikia fusiformis*), antioxidant peptide, cytoprotective function, molecular docking study, Keap1/Nrf2 pathway

## Abstract

Hijiki (*Hizikia fusiformis*) is a seaweed native to warm-temperate and subtropical regions that has a high edible value and economic value, with a production of about 2 × 10^5^ tons/year. Current research has clearly shown that the pharmacological activities of active ingredients from hijiki have covered a broad spectrum of areas, including antioxidant, hypoglycemic, antiviral, anticoagulant, anti-inflammatory, intestinal flora modulation, anti-aging, antineoplastic and antibacterial, and anti-Alzheimer’s disease areas. However, no studies have reported on the production of antioxidant peptides from hijiki proteins. The objectives of this study were to optimize the preparation process and explore the cytoprotective function and mechanisms of antioxidant peptides from hijiki protein. The results indicated that papain is more suitable for hydrolyzing hijiki protein than pepsin, trypsin, alkaline protease, and neutral protease. Under the optimized parameters of an enzyme dosage of 3%, a material–liquid ratio of 1:30, and an enzyme digestion time of 5 h, hijiki hydrolysate with a high radical scavenging activity was generated. Using ultrafiltration and serial chromatographic methods, ten antioxidant oligopeptides were purified from the papain-prepared hydrolysate and identified as DGPD, TIPEE, TYRPG, YTPAP, MPW, YPSKPT, YGALT, YTLLQ, FGYGP, and FGYPA with molecular weights of 402.35, 587.61, 592.64, 547.60, 532.53, 691.77, 523.57, 636.73, 539.58, and 553.60 Da, respectively. Among them, tripeptide MPW could regulate the Keap1/Nrf2 pathway to significantly ameliorate H_2_O_2_-induced oxidative damage of A549 cells by increasing cell viability and antioxidant enzyme (SOD, CAT, and GSH-Px) activity, decreasing ROS and MDA levels, and reducing the apoptosis rate. Molecular docking experiments show that HFP5 (MPW) exerts its inhibitory effect mainly through hydrogen bonds and hydrophobic interactions with the Kelch domain of the Keap1 protein, eventually facilitating the translocation of Nrf2 to the nucleus. Therefore, antioxidant peptides from hijiki can be applied to develop algae-derived health foods for treating diseases associated with oxidative stress.

## 1. Introduction

Since the inception of the free radical theory by the American scientist, Herman, there has been a profound understanding of the pivotal role played by excessive reactive oxygen species (ROS) in the intricate processes of aging and the onset of diverse diseases such as chronic infectious arthritis, neurodegenerative disorder, radiation damage, high blood pressure, cardiovascular diseases, and diabetes mellitus and its complications [1,2]. Utilizing powerful antioxidants is a valuable strategy to effectively eliminate excessive ROS within the body. However, the chemical antioxidants currently prevalent in production are associated with significant adverse effects, such as toxicity, potential risk of cancer, disruption of the body’s immune balance and the onset of gastrointestinal discomfort [3,4,5]. As a result, there is a growing emphasis on exploring safer and more natural alternatives to chemical antioxidants.

The antioxidant peptides from plant agricultural products and their processing by-products have garnered substantial global research interest [6]. Multiple cell models, animal models of human diseases, and oxidative stress tests were used to evaluate the efficacy of these peptides to combat ROS, mitigate oxidative injury, and regulate cell redox balances [7,8]. These studies have revealed that natural antioxidant peptides exhibit the capacity to reduce the production of ROS, enhance endogenous antioxidative defenses [9], and alleviate lipid peroxidation in different kinds of cells exposed to oxidative damage caused by a variety of oxidants [10].

Because the living environment is different from that of terrestrial organisms, marine organisms produce an abundance of proteins with special amino acid (AA) sequences to accommodate the high-salt, high-water pressure, low-light, low-temperature, and low-nutrient environment of the ocean. These special amino acid sequences may exhibit significant and diverse biological activities when they are hydrolyzed from parent proteins. Recently, marine active peptides have shown an intriguing antioxidant capacity for removing radicals, controlling lipid peroxidation, and regulating antioxidative defense systems [11,12]. For example, antioxidant peptides of LKPGN and LQP from Antarctic krill exhibited high radical scavenging abilities. Moreover, oligopeptides LKPGN and LQP from Antarctic krill can improve antioxidative enzyme activity to clear away superfluous ROS, increase the mitochondrial membrane potential (MMP), ameliorate DNA injury, and lower malondialdehyde (MDA) content to protect hepatocytes against oxidative damage [13]. PIISVYWK and FSVVPSPK from *Mytilus edulis* significantly improved the survival rate and lowered the elevated ROS level of H_2_O_2_-induced HUVECs disfunction by regulating the amount of Nrf2 into the nucleus to elevate HO-1 protein production, down-regulating the cleaved caspase-3 and Bax levels, inhibiting cytochrome C release, and restoring the MMP [14]. Three novel oligopeptides, LLVSeMY, MMDSeML and VSeMDSeML, were isolated from Se-enriched oyster hydrolysate, exhibited impressive cellular antioxidative functions, and significantly protected AAPH-injured HepG2 cells by removing surplus ROS and promoting antioxidative enzyme activity because they could regulate the Keap1/Nrf2 pathway by forming stable hydrogen and hydrophobic bonds with Keap1 [15].

Hijiki (*Hizikia fusiformis*) is a seaweed native to warm-temperate and subtropical regions and a common commercial algae with a production of approximately 2 × 105 tons/year. Current research has clearly shown that hijiki contains a variety of active ingredients, including polysaccharides, sterols, terpenoids, fucoidan polyphenols, amino acids, and trace elements [16,17,18]. Research on the pharmacological activities of these active ingredients has covered a broad spectrum of areas, including antioxidant, hypoglycemic, antiviral, anticoagulant, anti-inflammatory, intestinal flora modulation, anti-aging, antineoplastic, and antibacterial effects [19,20]. Recent studies have unequivocally demonstrated the significant impact of hijiki active ingredients in the treatment of Alzheimer’s disease [21]. Therefore, exploration of the active chemical composition of hijiki holds immense significance in facilitating its further development and utilization. However, no studies have investigated the production of antioxidant peptides from hijiki proteins. Thus, the purposes of this study are to optimize the preparation process of antioxidant peptides from hijiki proteins and explore their potential in mitigating oxidative stress in H_2_O_2_-damaged A549 cells. This study will provide a good starting point for further research on hijiki antioxidant peptides and the further development of new functional foods against oxidation-related diseases.

## 2. Materials and Methods

### 2.1. Materials, Reagents, and Instruments

Dried hijiki (*H. fusiformis*) was purchased from Dongtou Island (Wenzhou, China). Human lung cancer A549 cells and their specialized media were purchased from Procell Life Science & Technology Co., Ltd. (Wuhan, China). CCK-8 solution (10 mL) was purchased from Abbkine Scientific Co., Ltd. (Wuhan, China). Bicinchoninic acid assay (BCA) (500 times), hoechst 33258 staining solution (50 mL) and N-acetylcysteine (NAC) were purchased from Beyotime Biotechnology Co., Ltd. (Shanghai, China). Superoxide dismutase (SOD) (96 T), glutathione (GSH) (96 T), catalase (CAT) (100 T/96), malondialdehyde (MDA) (96 T), and reactive oxygen species (ROS) (100 T–500 T) were purchased from the Nanjing Jiancheng Bioengineering Research Institute (Nanjing, China). Ten oligopeptides, including DGPD, TIPEE, TYRPG, YTPAP, MPW, YPSKPT, YGALT, YTLLQ, FGYGP, and FGYPA, were synthesized by Apeptide Co., Ltd. (Shanghai, China).

### 2.2. Optimization of the Preparation Process of Hijiki Protein Hydrolysate

#### 2.2.1. Protease Screening

The dried hijiki was soaked, washed with distilled water, dried in an oven at 50 °C, ground into a powder, and processed with an 80-mesh sieve to remove large debris. After that, the resulting hijiki was submerged in 95% ethanol with a material–liquid ratio of 1:6 for 18 h to degrease, rinsed with distilled water, oven-dried at 50 °C, and stored at −20 °C.

Degreased hijiki was put in ultrapure water at a material–liquid ratio of 1:30 (*w*/*v*) and stirred for 0.5 h. Subsequently, the hijiki proteins in the mixed solution were degraded separately for 3 h by five proteases, including pepsin, trypsin, papain, alkaline protease, and neutral protease with a total enzyme concentration of 2.0% (m/v). Subsequently, the resulting solution was kept in 95 °C water bath for 15 min and centrifuged at 4500× *g* for 10 min to remove solid residues. The supernatants were lyophilized, and their DPPH· and HO· scavenging activities were determined according to the method described by Sheng et al. (2023) [22]. The papain-prepared hydrolysate showed the highest activity among five hijiki protein hydrolysates.

#### 2.2.2. Optimization of the Hydrolysis Process of Papain

Using DPPH· and HO· scavenging ratios as indicators, the hydrolysis parameters of papain, including the enzyme concentration (1%, 2%, 3%, 4%, and 5%, m/v), material–liquid ratio (1:20, 1:25, 1:30, 1:35, and 1:40), and enzymatic time (2, 3, 4, 5, and 6 h), were optimized. Finally, the hijiki protein hydrolysate generated under the optimized process was named HPH.

### 2.3. Preparation of Antioxidant Peptides from Hijiki Protein Hydrolysate (HPH)

#### 2.3.1. Ultrafiltration

To remove peptides with low activity, HPH was sequentially ultrafiltered using ultrafiltration membranes with pore sizes of 10, 5, 3, and 1 kDa, and five peptide fractions, including HPH-I (<1 kDa), HPH-II (1–3 kDa), HPH-III (3–5 kDa), HPH-IV (5–10 kDa), and HPH-V (>10 kDa), were prepared. HPH-I showed a higher antioxidative ability than HPH-I, HPH-II, HPH-III, HPH-IV, and HPH-V.

#### 2.3.2. Purification of Antioxidant Peptides from HPH-I Using Chromatography Methods

A HPH-I solution (50 mg/mL) was prepared, subjected to centrifugation, and treated with a 0.45 μm filter membrane to eliminate insoluble particles. After that, 5 mL of pretreated HPH-I solution was loaded in a DEAE-52 cellulose column (2.6 × 120 cm), and a gradient elution was carried out using 0, 0.05, 0.25, 0.5, 1, and 2 mol/L NaCl solutions at 3 mL/min. Eight peptide fractions were collected according to the chromatographic curve of HPH-I at 220 nm and named as IF-I, IF-II-1, IF-II-2, IF-II-3, IF-III, IF-IV, IF-V, and IF-VI. Their radical scavenging rates at 4.0 mg/mL were assessed, and peptide components with superior antioxidant activities (IF-III) were selected for subsequent gel chromatography purification stages.

An IF-III solution (50 mg/mL) was treated with a 0.45 μm filter membrane to eliminate insoluble particles. A pretreated IF-III solution (5 mL) was loaded in a Sephadex G-15 column (1.6 × 100 cm), eluted by distilled water at 0.6 mL/min, and determined at 220 nm. Finally, only one peptide fraction (IF-IIIa) was meticulously collected from IF-III at 220 nm. The purified fraction (IF-IIIa) was freeze-dried to evaluate its DPPH· and HO· scavenging ratios.

IF-IIIa was treated with a 0.45 μm filter membrane to eliminate insoluble particles and subsequently separated by the Zorbax SB C-18 column (4.6 × 250 mm, 5 µm) in the Agilent 1200 system. The sample was eluted by 45% acetonitrile at 1.0 mL/min. The elution solution was detected at 220 nm. In the end, ten antioxidative peptides (HFP1 to HFP10) were separated from IF-IIIa.

### 2.4. Peptide Sequence Analysis and Molecular Weight (MW) Determination

The sequences of ten peptides (HFP1 to HFP10) were identified by an Applied Biosystems 494 protein/peptide sequencer (Applied Biosystems, Foster City, CA, USA). The MWs of ten peptides (HFP1 to HFP10) were measured by an ESI-MS (SHIMADZU LCMS-2020) (Dongjing, Japan). An in vitro antioxidant capacity test confirmed that HFP5 (MPW) (Met-Pro-Trp) had the best clearance rate of DPPH· and HO·, so HFP5 (MPW) was selected for the next experiment.

### 2.5. Cytoprotection of Antioxidant Peptide (HFP5) on H_2_O_2_-Damaged A549 Cells

#### 2.5.1. Establishment of H_2_O_2_-Damaged Model of A549 Cells

A549 cells (8 × 10^3^ cells/well) were seeded into a 96-well plate which contained 180 μL of culture media. After 24 h of incubation, H_2_O_2_ (20 μL) was added to the HFP5 (MPW) groups with a final concentration of 100, 200, 300, 400, and 500 μM, respectively. Following a 4 h incubation period, the liquid in the 96-well plate was removed and a CCK-8 solution (CCK-8 to culture medium ratio of 1:10, 200 μL) was supplemented. After 45 min of incubation in darkness, the absorbance (A_450nm_) was measured at 450 nm to evaluate the cell viability and determine the optimal concentration of H_2_O_2_ for reducing the oxidative damage in A549 cells [23].

Cell viability (%) = (A450 nm (sample)/A450 nm (control)) × 100(1)

#### 2.5.2. Impact of the Antioxidant Peptide (HFP5) on the Viability of A549 Cells

After A549 cells were incubated for 24 h, 20 μL of the peptide (HFP5) was added to the sample group, and the final concentrations of HFP5 (MPW) were 0, 50, 100, 200, 400, and 800 μM, respectively. After 4 h of incubation, the viability of A549 cells in each group was determined. In addition, the same volume of a PBS (pH 7.2) and NAC solution (1.0 mmol/L) was used instead of a HFP5 (MPW) solution in the control and positive group.

#### 2.5.3. Cytoprotection of Antioxidant Peptide (HFP5) on the H_2_O_2_-Damaged Cell Model

After A549 cells were incubated for 24 h, 20 μL of the PBS and NAC solution (1.0 mmol/L) and HFP5 (MPW), with previously defined concentrations, were separately put in the groups of the control, model, positive control, and HFP5. After being cultured for 24 h, 20 μL of H_2_O_2_ (400 μM) was added in the groups of the positive control and HFP5 (MPW). After 4 h of incubation, the viability of the A549 cells in each group was determined.

#### 2.5.4. Determination of the Levels of ROS, SOD, CAT, GSH-Px, and MDA

The intracellular level of ROS was measured using the method described by Zheng et al. (2024) [2]. In short, A549 cells were pretreated with HFP5 (MPW) at 100, 200 and 400 μM. After 12 h of incubation, A549 cells were incubated with 400 μM H_2_O_2_. After 2 h of incubation, we used a PBS (pH 7.0, 0.02 M) to wash the A549 cells of each group, and the treated A549 cells were incubated with 2′,7′-Dichlorofluorescein diacetate (DCFH2-DA) (10 μM). After 30 min of incubation, the content of the ROS in each group was determined and expressed as a percentage of control.

The production of MDA and the activities of SOD, GSH, and CAT were determined using assay kits according to the protocols of the manufacturer [24].

#### 2.5.5. The Morphological Observation of A549 Cells

According to the method described in 2.5.3, A549 cells were separately treated with HFP5 (MPW), NAC, and H_2_O_2_. After that, Hoechst 33342 (0.1 mL) was added into the A549 cells of each group. After 30 min of incubation, the solution in each group was removed and the A549 cells were washed three times using a PBS. After that, the morphology of the A549 cells was photographed and the fluorescence intensity was measured using ImageJ-win64 software.

### 2.6. Molecular Docking Experiment

The crystal structure of Keap1 was acquired from the PDB database (PDB ID: 2FLU). According to the previous studies [8], the C-terminal Kelch domain of Keap1 was selected to investigate the possible binding mode of HFP5 (MPW) to Keap1. A molecular docking analysis of HFP5 (MPW) was performed in the Kelch pockets of Keap1 using AutoDock Vina. At default settings, the affinity of HFP5 (MPW) to Keap1 was acquired by a docking analysis of AutoDock Vina and Pymol. Discovery Studio was applied to visualize the docking results, including 2D and 3D plots.

### 2.7. Data Statistics

All experimental data are reported as the mean ± SD (*n* = 3). An ANOVA test was used to analyze the experimental data, and the significant differences in each group (*p* < 0.05, *p* < 0.01, or *p* < 0.001) were analyzed by a Duncan’s multiple range test.

## 3. Results

### 3.1. Optimization of the Preparation Process of Hijiki Protein Hydrolysate

#### 3.1.1. Screening of Protease Types

**Figure 1:**
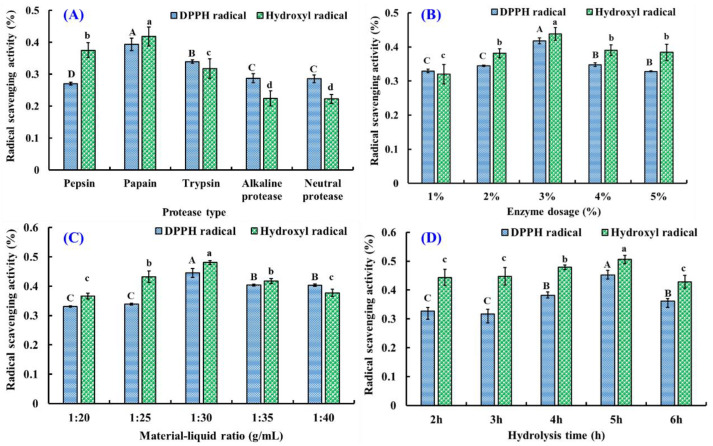
Effects of the protease type (**A**), enzyme dosage (**B**), material–liquid ratio (**C**), and hydrolysis time (**D**) on the DPPH· and HO· scavenging rates of hijiki protein hydrolysates at 4.0 mg/mL. ^a–d^ or ^A–C^; same letter indicates no significant difference (*p* > 0.05).

In order to screen the suitable protease, hijiki proteins were separately degraded by pepsin, trypsin, papain, alkaline protease, and neutral protease, and the radical scavenging ratios of the prepared hydrolysates were determined and are depicted in Figure 1A. The DPPH· and HO· scavenging ratios of papain-prepared hydrolysate at 4.0 mg/mL were 39.4% ± 1.9% and 41.9% ± 3.0%, respectively, which was observably higher than those of hydrolysates prepared by pepsin, trypsin, alkaline protease, and neutral protease (*p* < 0.05). As a result of the screening process, papain was selected for subsequent investigations in this study.

#### 3.1.2. Optimization of Hydrolysis Process of Hijiki Proteins by Papain

Figure 1B–D depicts the influence of the enzymatic parameters of papain on the DPPH· and HO· scavenging rates of hijiki protein hydrolysates. At 3% enzyme dosage, the DPPH· and HO· scavenging ratios of hijiki protein hydrolysate at 4.0 mg/mL were 41.8% ± 0.9% and 43.8% ± 1.9%, respectively, which were dramatically larger than the ratios of hydrolysates produced at four other papain concentrations (Figure 1B). Figure 1C indicates that the material–liquid ratio significantly influences the antioxidative ability of hijiki hydrolysates, and the highest DPPH· (44.5% ± 1.6%) and HO· (48.1% ± 0.7%) scavenging activities of the produced hydrolysate were achieved with a material–liquid ratio of 1:30. Additionally, the maximum DPPH· and HO· scavenging rates of hijiki protein hydrolysate were 45.2% ± 1.5% and 50.6% ± 1.5% when the hijiki protein was hydrolyzed by papain for 5 h (Figure 1D). Eventually, an enzyme dosage of 3%, a material–liquid ratio of 1:30, and a hydrolyzed time of 5 h were selected as the optimal parameters of papain to hydrolyze hijiki proteins, and the hijiki hydrolysate was generated under the optimized parameters and named as HPH.

#### 3.1.3. Purification of the Antioxidant Peptides from Hijiki Protein Hydrolysate (HPH)

**Figure 2:**
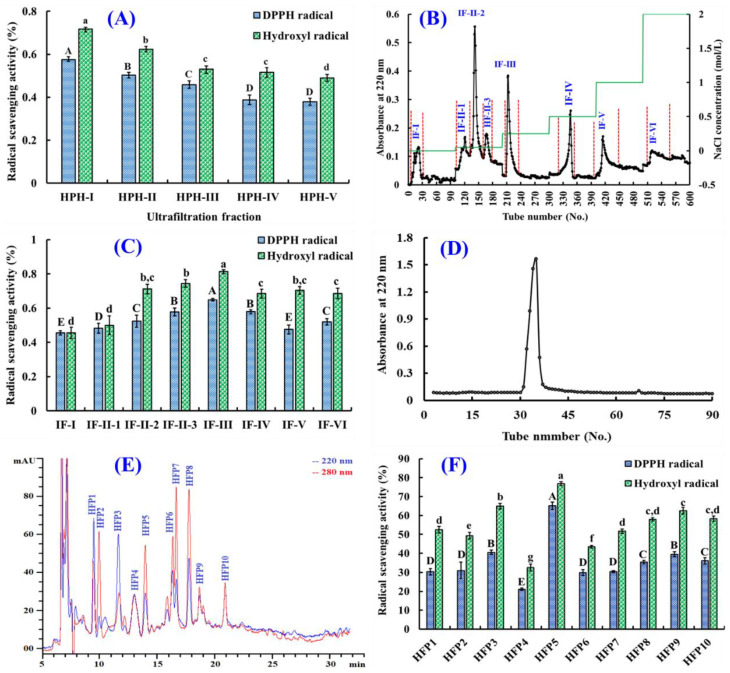
The purification of antioxidant peptides (HFP1–HFP10) from hijiki protein hydrolysate (HPH). (**A**) The radical scavenging activity of five ultrafiltration fractions (HPH-I ~ HPH-V) from HPH; (**B**) the elution profile of the ultrafiltration fraction (HPH-I) on a DEAE-52 cellulose column; (**C**) the radical scavenging ratios of HPH-I’s peptide fractions (IF-I ~ IF-VI); (**D**) the elution profile of peptide subfraction (IF-III) on a Sephadex G-15 column; (**E**) the elution profile of peptide subfraction (IF-IIIa) separated by RP-HPLC; (**F**) The radical scavenging ratios of peptides (HFP1–HFP10) from IF-IIIa. ^a–g^ or ^A–E^ Same letter indicates no significant difference (*p* > 0.05).

Through membrane ultrafiltration technology, HPH was divided into five peptide fractions, including HPH-I, HPH-II, HPH-III, HPH-IV, and HPH-V, and their radical scavenging rates are depicted in Figure 2A. These results suggested that HPH-I had the strongest activity among five fractions, with DPPH· and HO· scavenging rates of 57.61% ± 1.38% and 71.76% ± 2.35%, respectively. The rates of HPH-I were significantly greater than those of HPH (48.09% ± 1.36% and 58.76% ± 2.97%), HPH-II (50.35% ± 1.17% and 62.33% ± 1.39%), HPH-III (45.83% ± 1.88% and 53.03% ± 1.61%), HPH-IV (38.67% ± 2.30% and 51.67% ± 2.03%), and HPH-V (37.92% ± 1.67% and 48.97% ± 1.69%) (*p* < 0.05). Then, HPH-I was chosen for subsequent isolation to prepare antioxidant peptides.

Using a DEAE-52 cellulose column, HPH-I was fractionated into eight subfractions (IF-I, IF-II-1, IE-II-2, HF-II-3, IF-III, IF-IV, IF-V, IF-VI) according to its 220 nm chromatographic diagram (Figure 2B). The DPPH· and HO· scavenging rates of IF-III at 4.0 mg/mL were 57.75% ± 0.65% and 74.44% ± 1.12%, which were remarkably greater than those of seven other peptide fractions (*p* < 0.05) (Figure 2C). Subsequently, IF-III was purified by a Sephadex G-15 column (Figure 2D), but only one peptide fraction (IF-IIIa) was obtained at 220 nm. At 4.0 mg/mL, the DPPH· and HO· scavenging ratios of IF-IIIa were 63.12% ± 2.06% and 79.51% ± 2.46%, which were remarkably greater than those of IF-III (*p* < 0.05).

A peptide fraction of IF-IIIa with a strong radical scavenging capability was ultimately purified by RP-HPLC, and its peptide profile is depicted in Figure 2E. Then, ten peptides with retention times (RTs) of 9.57 (HFP1), 10.06 (HFP2), 11.77 (HFP3), 13.03 (HFP4), 14.01 (HFP5), 16.45 (HFP6), 16.81 (HFP7), 17.80 (HFP8), 18.79 (HFP9), and 20.95 min (HFP10) were isolated, collected, and lyophilized. In addition, ten peptides (HFP1–HFP10) purified from IF-IIIa were further enriched for activity evaluation and structure identification.

### 3.2. Structural Identification and Radical Scavenging Activity of Purified Antioxidant Peptides (HFP1–HFP10)

**Figure 3:**
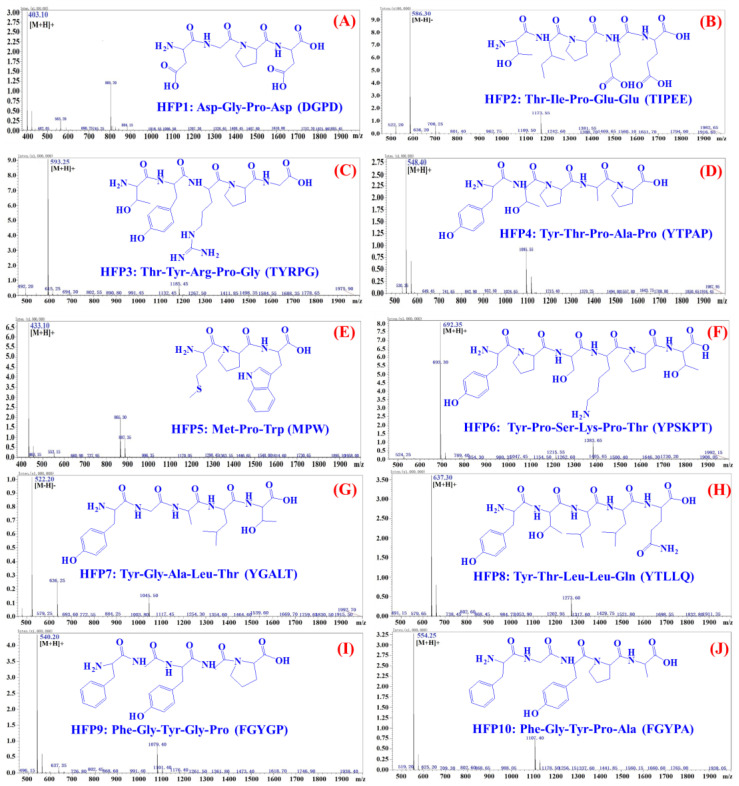
Mass spectra of ten isolated peptides (HFP1–HFP10) from hijiki protein hydrolysate (HPH). (**A**) HFP1; (**B**) HFP2; (**C**) HFP3; (**D**) HFP4; (**E**) HFP5; (**F**) HFP6; (**G**) HFP7; (**H**) HFP8; (**I**) HFP9; (**J**) HFP10.

Using a Protein/Peptide Sequencer and ESI-MS, the amino acid sequences of ten antioxidant peptides (HFP1–HFP10) were identified as Asp-Gly-Pro-Asp (DGPD, HFP1), Thr-Ile-Pro-Glu-Glu (TIPEE, HFP2), Thr-Tyr-Arg-Pro-Gly (TYRPG, HFP3), Tyr-Thr-Pro-Ala-Pro (YTPAP, HFP4), Met-Pro-Trp (MPW, HFP5),Tyr-Pro-Ser-Lys-Pro-Thr (YPSKPT, HFP6), Tyr-Gly-Ala-Leu-Thr (YGALT, HFP7), Tyr-Thr-Leu-Leu-Gln (YTLLQ, HFP8), Phe-Gly-Tyr-Gly-Pro (FGYGP, HFP9), and Phe-Gly-Tyr-Pro-Ala (FGYPA, HFP10) with MWs of 402.35, 587.61, 592.64, 547.60, 532.53, 691.77, 523.57, 636.73, 539.58, and 553.60 Da, respectively (Figure 3). The determined MWs were consistent with their theory MWs of 402.36 Da (HFP1), 587.62 Da (HFP2), 592.64 Da (HFP3), 547.60 Da (HFP4), 532.54 Da (HFP4), 691.77 Da (HFP6), 523.58 Da (HFP7), 636.74 Da (HFP8), 539.58 Da (HFP9), and 553.61 Da (HFP10).

As illustrated in Figure 2F, The DPPH· and HO· scavenging rates of HFP5 (MPW) at 2.0 mg/mL were 65.12% ± 1.96% and 76.84% ± 1.08%, which were remarkably greater than those of nine other separated peptides (*p* < 0.05). Therefore, HFP5 was selected for the subsequent cytoprotective experiment on H_2_O_2_-damaged A549 cells.

### 3.3. Cytoprotective Effect of HFP5 (MPW) on H_2_O_2_-Damaged A549 Cells

#### 3.3.1. Effect of H_2_O_2_ and HFP5 (MPW) on Viability of A549 Cells

**Figure 4:**
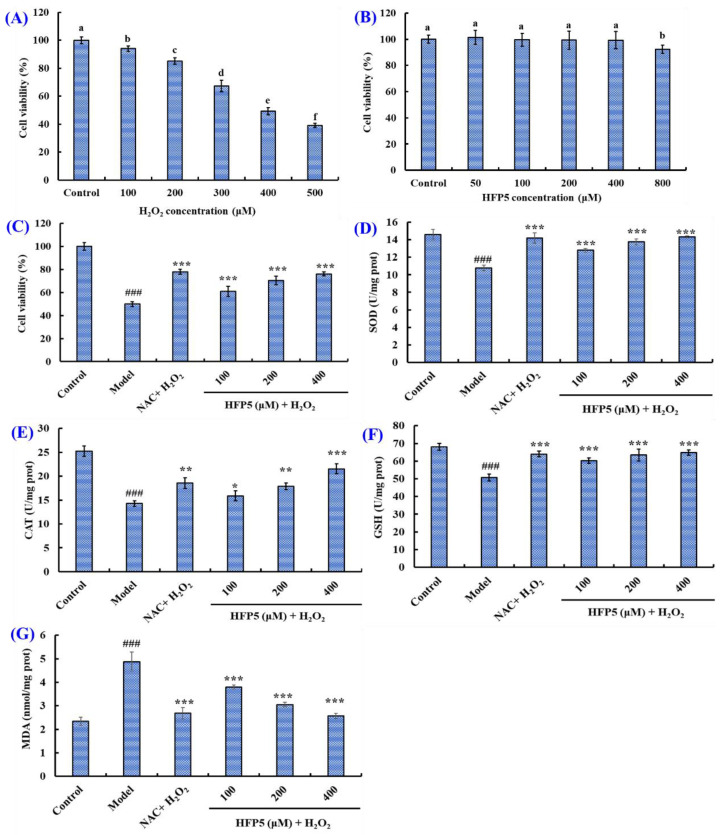
Effects of H_2_O_2_ (**A**) and HFP5 (**B**) on the cell viability of A549 cells; cytoprotective effect (**C**) of HFP5 on H_2_O_2_-induced A549 cells; effects of HFP5 on the SOD (**D**), GSH-Px (**E**), CAT (**F**), and MDA (**G**) levels of H_2_O_2_-induced A549 cells. (**A**,**B**): ^a–f^ Same letter indicates no significant difference (*p* > 0.05); (**C**–**G**) ^###^ *p* < 0.001 vs. control group; * *p* < 0.05, ** *p* < 0.01 and *** *p* < 0.001 vs. model group.

To assess the cytoprotective ability of HFP5 (MPW) on H_2_O_2_-damaged A549 cells, the effect of H_2_O_2_ concentration (0–500 μM) on the viability of A549 cells was analyzed. Figure 4A depicts the viability of A549 cells, revealing a significant decline trend when the H_2_O_2_ concentration is increased gradually from 0 to 500 μM (*p* < 0.05). In addition, the cell viability was 49.26 ± 2.49% when the H_2_O_2_ concentration was 400 μM. The previous literature has reported that a H_2_O_2_ concentration which results in a decrease of about 50% cell viability is suitable for establishing the oxidativedamaged cell model [5]. Therefore, the H_2_O_2_ concentration of 400 μM was selected for establishing the oxidative-damaged model of A549 cells.

Figure 2F indicates that HFP5 (MPW) showed high scavenging activity in regard to DPPH· and HO· among ten prepared peptides (HFP1 to HFP10). Then, the effect of HFP5 at 0–800 μM on the viability of A549 cells was evaluated (Figure 4B), and the viability of HFP5-treated A549 cells ranged from 92.37 ± 3.17% to 101.42 ± 5.29%. However, the cell viability of HFP5-treated A549 cells at 800 μM was significantly lower than those of HFP5-treated A549 cells at other concentrations (0–400 μM). Therefore, we chose HFP5 for subsequent experiments at concentrations of 100, 200, and 400 μM.

Figure 4C presents the cytoprotective effect of HFP5 at 100–400 μM on H_2_O_2_-damaged A549 cells. The viability of A549 cells in the model group was dramatically reduced to 49.92% ± 1.92% (*p* < 0.001), but the viability of HFP5-treated A549 cells at 100, 200, and 400 μM were 61.02% ± 4.24%, 70.37% ± 3.65% and 76.11% ± 1.69%, respectively, and the viability was remarkably greater than those of the model group (*p* < 0.001), but slightly less than that of positive control group (77.84% ± 2.16%). The present results indicated that HFP5 had a high cytoprotective function on H_2_O_2_-damaged A549 cells.

#### 3.3.2. Effect of HFP5 on the Levels of ROS, SOD, GSH-Px, CAT, and MDA in H_2_O_2_-Induced A549 Cells

The role of SOD, GSH-PX, and CAT is integral to the overall defense strategy of the intracellular antioxidant system, especially in terms of scavenging ROS from the body [25]. The effect of HFP5 on the activity of SOD, GSH-Px, and CAT at 100, 200, and 400 μM were depicted in Figure 4D–F. In the model group, the SOD, GSH-Px, and CAT activities were 10.78 ± 0.32, 50.71 ± 1.9, and 14.3 ± 0.59 U/mg prot, respectively, which is remarkably less than those in the control group. However, HFP5 treatment significantly improved the activity of SOD, GSH-Px, and CAT in H_2_O_2_-damaged A549 cells (*p* < 0.05). At 400 μM, the SOD, GSH-Px, and CAT activity correspondingly increased to 14.32 ± 0.11, 64.74 ± 1.52, and 21.56 ± 1.05 U/mg prot.

MDA is one of the end products of the intracellular lipid peroxidation of PUFAs and is often considered as a key marker of intracellular oxidative stress and antioxidant status [26]. Figure 4G indicates that H_2_O_2_ treatment significantly increased the MDA content in A549 cells from 2.35 ± 0.17 nmol/mg prot to 4.88 ± 0.41 nmol/mg prot. However, the MDA content in H_2_O_2_-treated A549 cells was significantly decreased to 3.8 ± 0.09, 3.05 ± 0.1, and 2.57 ± 0.11 nmol/mg prot after treatment with 100, 200, and 400 μM of HFP5.

#### 3.3.3. Effect of HFP5 on the ROS Level of H_2_O_2_-Induced A549 Cells

**Figure 5:**
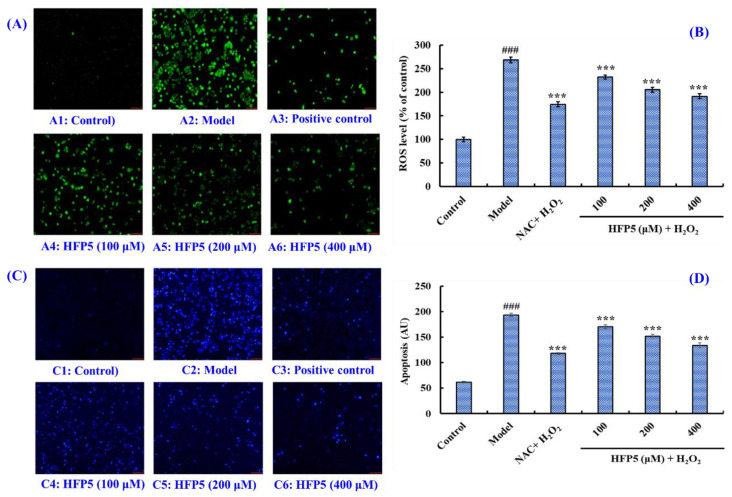
Analysis of the ROS content in A549 cells by the DCFH-DA staining method (**A**); effect of HFP5 at 100–400 μM on the content of ROS in H_2_O_2_-damaged A549 cells (**B**); apoptosis analysis of HFP5 on H_2_O_2_-damaged A549 cells by a Hoechst 33,342 staining assay (**C**); effect of HFP5 at 100–400 μM on the apoptosis rate of H_2_O_2_-damaged A549 cells (**D**). ^###^ *p* < 0.001 vs. control group; *** *p* < 0.001 vs. model group.

Overexposure to UV radiation, chronic stress conditions, vigorous physical exercise, and an inappropriate diet can result in the overproduction of intracellular ROS, which produces chemical modifications and has destructive effects in regard to proteins, PUFAs, carbohydrates, and DNA, contributing to the development of many ROS-mediated diseases related to the digestive, respiratory, and nervous systems [27,28]. Figure 5A,B reveal the effect of HFP5 on the ROS levels of H_2_O_2_-treated A549 cells. Figure 5A shows that H_2_O_2_ treatment remarkably heightened the fluorescence intensity and area of A549 cells, demonstrating that the ROS content in H_2_O_2_-treated A549 cells was 2.86-fold of the control group (Figure 5B). Moreover, the NAC (Figure 5(A3)) and HFP5 groups (Figure 5(A4–A6)) could significantly decrease the fluorescence area and intensity of H_2_O_2_-treated A549 cells, demonstrating a high reducibility to intracellular ROS. Compared with the model group (268.3% ± 6.21%), the ROS levels of H_2_O_2_-treated A549 cells were significantly decreased by all three concentrations of HFP5 treatment. The ROS levels were 232.2% ± 4.24%, 205.3% ± 5.41%, and 191.4% ± 5.1% at HFP5 concentrations of 100, 200, and 400 μM, respectively *(p* < 0.001) (Figure 5B).

#### 3.3.4. Effect of HFP5 on Apoptosis of H_2_O_2_-Induced A549 Cells

A Hoechst 33342 staining experiment (Figure 5C) depicted the HFP5 treatment significantly decreasing the blue fluorescence intensity of H_2_O_2_-damaged A549 cells; however, it also indicated that HFP5 treatment could not restore H_2_O_2_-damaged A549 cells to an undamaged state. The finding illustrated that HFP5 could strongly protect H_2_O_2_-damaged A549 cells, which agreed with the results of Figure 3C. Therefore, we further quantitatively analyzed the effect of HFP5 on the apoptosis of H_2_O_2_-damaged A549 cells using ImageJ-win64 software (Figure 5D). When the HFP5 concentration was 100, 200, and 400 μM, the apoptosis rate of the A549 cells was significantly decreased from 193.70 ± 3.3 AU to 170.78 ± 3.76, 151.62 ± 3.87, and 133.55 ± 5.27 AU in the model group, respectively (*p* < 0.001). The data indicated that 100–400 μM of HFP5 concentration-dependently inhibited the apoptosis of H_2_O_2_-induced A549 cells.

### 3.4. Molecular Docking Analysis of HFP5 (MPW) with Keap1 Protein

**Figure 6:**
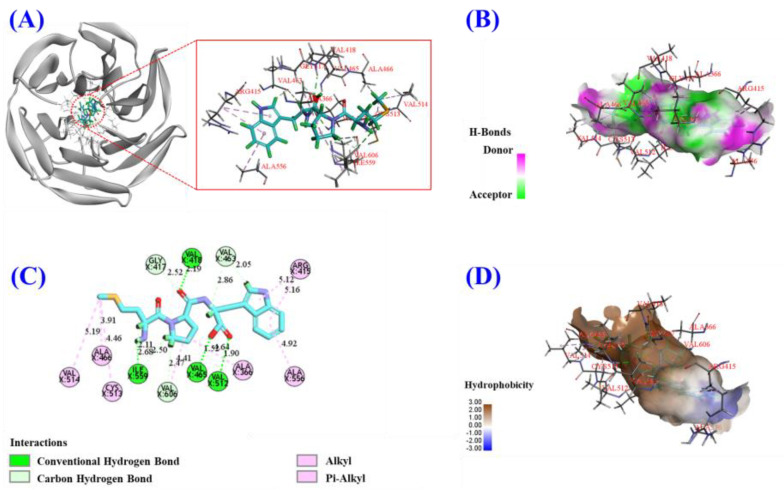
The molecular docking result of HFP5 (MPW) with Keap1 protein. (**A**) A 3D detailed map of the best binding conformation using Pymol; (**B**) a 2D map of molecular interactions using DS; (**C**) a 3D hydrogen bonding interaction surface map using DS; (**D**) a 3D hydrophobic interaction surface map using DS.

The Kelch domain of Keap1 can interact with the Neh2 domain of Nrf2. Therefore, other compounds occupying the Kelch domain can promote the entry of Nrf2 into the nucleus, thus further initiating the Nrf2 pathway [29,30]. Figure 6 depicts the molecular docking result of HFP5 (MPW) with Keap1 protein. The affinity of HFP5 (MPW) with Keap1 protein was −9.0 Kcal/mol, indicating that HFP5 (MPW) and Keap1 protein could bind tightly together. In addition, HFP5 (MPW) formed hydrogen bonds with Keap1’s amino acid residues of Gly417, Val418, Val463, Ile559, Val606, Val465, and Val512 and also formed hydrophobic interactions with Arg415 (P1), Val514, Ala466, Cys513, Ala 366, and Ala556 (P3). Therefore, HFP5 (MPW) exerts its inhibitory effect mainly through hydrogen bonds and hydrophobic interactions with the Kelch domain of Keap1 protein, including the active pockets of P1 and P3, and it eventually facilitates the translocation of Nrf2 to the nucleus.

## 4. Discussion

### 4.1. Optimization of the Preparation Process of Hijiki Protein Hydrolysate

The function of hydrolysates from dietary proteins to facilitate human health has exceeded the previously recognized nutritional value. Studies have shown that dietary protein hydrolysates have many functions, such as antioxidant and anti-inflammatory elements, lowering blood pressure, and regulating blood sugar. These pharmacological or physiological functions are inseparable from bioactive peptides generated from dietary proteins by different hydrolysis treatments [31,32,33]. In addition, enzymatic hydrolysis is the best choice for food processing due to its safety and easily controlled hydrolysis conditions [31,34]. Currently, there are two popular methods for preparing active hydrolysates: single-enzyme hydrolysis and complex-enzyme hydrolysis. In this study, hijiki proteins were hydrolyzed by single-enzyme hydrolysis to produce HPH, and, among the five protease hydrolysates, the papain hydrolysate of hinoki proteins showed the strongest free radical scavenging ability (Figure 1A), which is consistent with previous studies that indicated that enzyme specificity significantly affects the structure of peptides, which is related to peptides’ pharmacological activities [35,36,37].

### 4.2. The Structural Identification and Radical Scavenging Activity of Purified Antioxidant Peptides (HFP1–HFP10)

In general, two factors, including MW and amino acids, are thought to have the greatest influence on the pharmacological functions of bioactive peptides [31,38]. Generally, the smaller the MWs of the peptides, the better their binding affinity to the target molecules, thus enhancing their antioxidant ability [39,40]. In addition, Sila and Bougatef (2016) reported that low average MWs positively affect the antioxidative activity of protein hydrolysate from marine by-products [41]. In the experiment, 10 antioxidant oligopeptides (HFP1 to HFP10), the molecular weight of which were 402.35, 587.61, 592.64, 574.6, 432.53, 691.77, 523.57, 636.73, 539.58 and 553.6 Da, respectively, are linked to three peptides, tetrapeptide, or pentapeptide. Moreover, HFP5 (MPW) is a tripeptide compound with a MW of 402.35 Da, which greatly helps it to approach and effectively bind to the target molecules, exerting its antioxidant effects.

Hydrophobic/aromatic amino acids, such as Trp, Pro, Met, Ile, and Leu, are considered the key amino acids of antioxidant peptides. These amino acids can not only act as proton or hydrogen donors to give play to the antioxidant function but also improve the solubility of peptides in lipids and accelerate the binding of peptides and radicals [41,42]. Wei et al. (2024) found that Val, Pro, Met, and Tyr enhance the antioxidative properties of VPPP and MYP [38]. Similarly, Pro is considered to play an important role in the antioxidant effects of AVPYPQR and VLPVPQK [43]. In addition, Lin et al. (2024) reported that the functional groups of hydrophobic/aromatic amino acids, such as the pyrrole ring in Pro, the benzene ring in Trp and Phe, and the methylthio group (-S-CH3) in Met, contributed to the strong radical scavenging, reducing the power and metal chelating capability of the peptides [44]. Thus, HFP5 (MPW) exhibits the typical structural features of the above-mentioned antioxidant peptides, especially the three key amino acids of Met, Pro, and Trp.

### 4.3. Cytoprotective Effect of HFP5 (MPW) on H_2_O_2_-Damaged A549 Cells

The accumulation of ROS in cells can cause oxidative stress, which causes cell necrosis and apoptosis and is closely associated with diabetes and neurodegenerative, heart, and liver diseases [24,45]. In this study, HFP5 (MPW) showed a protective effect against H_2_O_2_-induced oxidative stress in A549 cells. HFP5 (MPW) at a 100–400 μM concentration can significantly reduce the contents of ROS (Figure 5A,B) and MDA (Figure 4G) in A549 cells under H_2_O_2_-induced oxidative stress, significantly enhance the activities of intracellular antioxidant enzymes (GSH, SOD, CAT) (Figure 4D–F), and inhibit the H_2_O_2_-induced apoptosis of A549 cells (Figure 5C,D). It was proven that HFP5 (MPW) had a strong antioxidant capacity and protective effect on cells under oxidative stress.

### 4.4. Molecular Docking Analysis of HFP5 (MPW) with Keap1 Protein

In normal conditions, Nrf2 undergoes ubiquitination and degradation in the cytoplasm by interacting with Keap1. However, in response to stress, Nrf2 disassociates from Keap1, translocates to the nucleus, and interacts with ARE, thereby initiating the Nrf2 pathway to promote the expression of detoxification and antioxidant enzymes such as HO-1, GSH, SOD, and CAT [23,46]. Molecular docking experiments (Figure 6) showed that HFP5 (MPW) has a high affinity with Keap1 protein and exerts its inhibitory effect mainly through hydrogen bonding and hydrophobic interactions with the Kelch domain of Keap1 protein (including the active pockets of P1 and P3); additionally, it also promotes the translocation of Nrf2 to the nucleus, thereby activating the Nrf2 pathway, which protects A549 cells from H_2_O_2_-induced oxidative stress.

Therefore, natural antioxidant peptides have been widely concerned with preventing and ameliorating oxidative damage by regulating oxidative stress responses. LAWY, LPGCP, and VSRRFIYYL from fermented broad-bean paste can initiate the Keap1/Nrf2 pathway and protect HepG2 cells from AAPH-induced oxidative damage through eliminating excess ROS and MDA [44]. Wu et al. (2023) reported that ARW, YPAGP, and DPAGP can regulate AMPK/Nrf2 pathways to suppress lipid accumulation and oxidative damage in FFA-induced HepG2 cells [8]. Seahorse peptide (SHP) can increase the levels of CAT and SOD to remarkably increase cell viability and lower ROS production and apoptotic DNA damage in AAPH-induced Vero cells. Furthermore, SHP treatment significantly lowered cell mortality, ROS generation, and lipid peroxidation in zebra fish embryos [47]. Antioxidant peptides of YDYD, ARW, and DDGGK from monkfish swim bladders displayed the dramatic ability to inhibit lipid peroxidation and Ferric-reducing powers. Furthermore, YDYD and ARW can defend plasmid DNA and HepG2 cells from oxidative damage [22].

## 5. Conclusions

In this study, the process of preparing antioxidant peptides from hijiki was optimized, and ten antioxidant peptides were purified from the resulting protein hydrolysate (HFP) using a combination of ultrafiltration and chromatography methods; these peptides were identified as DGPD, TIPEE, TYRPG, YTPAP, MPW, YPSKPT, YGALT, YTLLQ, FGYGP, and FGYPA, respectively. MPW, with the highest radical scavenging activity, exhibited a cytoprotective function in regard to H_2_O_2_-damaged A549 cells because it can concentration-dependently enhance the SOD, GSH-Px, and CAT activity to improve cell viability, reduce apoptotic rate, and lower MDA and ROS production. A molecular docking study suggested that MPW can regulate Nrf2 pathways by occupying the Kelch domain of Keap1 to Nrf2 to exert its antioxidant function. In addition, the antioxidant peptides from hijiki (*H. fusiformis*) showed a significant protective effect on the oxidation-damaged cells, which will facilitate the development of algae-derived health products for the treating of oxidative injury-related diseases.

## Data Availability

The original contributions presented in the study are included in the article; further inquiries can be directed to the corresponding author.
